# SAGA DUB-Ubp8 Deubiquitylates Centromeric Histone Variant Cse4

**DOI:** 10.1534/g3.115.024877

**Published:** 2015-11-25

**Authors:** Claudia Canzonetta, Stefano Vernarecci, Michele Iuliani, Cristina Marracino, Claudia Belloni, Paola Ballario, Patrizia Filetici

**Affiliations:** * Institute Pasteur Fondazione-Cenci Bolognetti, Sapienza University of Rome, 00185, Italy; † Institute of Molecular Biology and Pathology-National Research Council, Sapienza University of Rome, 00185, Italy; ‡ Department of Biology and Biotechnology “C. Darwin”, Sapienza University of Rome, 00185, Italy

**Keywords:** SAGA complex, DUB-Ubp8, deubiquitylation, histone variant Cse4, centromere, mitotic stability

## Abstract

Aneuploidy, the unbalanced segregation of chromosomes during cell division, is recurrent in many tumors and the cause of birth defects and genetic diseases. Centromeric chromatin represents the chromosome attachment site to the mitotic spindle, marked by specialized nucleosomes containing a specific histone variant, CEN-H3/Cse4, in yeast. Mislocalization of Cse4 outside the centromere is deleterious and may cause aberrant chromosome behavior and mitotic loss. For this reason, ubiquitylation by the E3-ubiquitin ligase Psh1 and subsequent proteolysis tightly regulates its restricted localization. Among multiproteic machineries, the SAGA complex is not merely engaged in acetylation but also directly involved in deubiquitylation. In this study, we investigated the role of SAGA-DUB’s Ubp8-driven deubiquitylation of the centromeric histone variant Cse4 in budding yeast. We found that Ubp8 works in concert with the E3-ubiquitin ligase Psh1, and that its loss causes defective deubiquitylation and the accumulation of a short ubiquitin oligomer on Cse4. We also show that lack of Ubp8 and defective deubiquitylation increase mitotic instability, cause faster Cse4 proteolysis and induce mislocalization of the centromeric histone outside the centromere. Our data provide evidence for a fundamental role of DUB-Ubp8 in deubiquitylation and the stability of the centromeric histone in budding yeast.

The SAGA (Spt-Ada-Gcn5-acetyltransferase) complex is a key multicomponent regulator of acetylation. Through its deubiquitination (DUB) module it is also involved in deubiquitylation exerted, for example, on histone H2B (Henry *et al.* 2003; Daniel *et al.* 2004). The ubiquitin protease Ubp8 (ubiquitin-specific processing protease 8) performs a discrete function in the DUB module (Ingvarsdottir *et al.* 2005). Its deubiquitylation activity is directly involved in the transcriptional activation of SAGA-responsive genes, in nucleosome eviction and remodelling, and it is often associated with concurrent histone H3 methylation (Shukla *et al.* 2006). Also, the SAGA DUB module (Sgf73, Sgf11, Sus1, Ubp8) has been shown to couple transcription with mRNA export (Kohler *et al.* 2006; Klockner *et al.* 2009). Among the Ubp8 orthologs, *Drosophila melanogaster* Non-stop is involved in axon guidance in the optic lobe (Weake *et al.* 2008), and human USP22 represents a signature associated with poor prognosis in various human cancers (Zhang *et al.* 2008). Also, USP22 silencing promotes apoptosis and cell cycle arrest in human brain gliomas (Li *et al.* 2013). Taken together, these multifunctional examples demonstrate the biological relevance of the SAGA complex and its DUB in cellular differentiation and human disease.

Chromosomal regions such as centromeres are substrates of epigenetic modifications. Although chromatin modifiers have been mainly linked to the regulation of gene expression, they are also likely to be engaged in the regulation of key processes in mitosis and meiosis. Centromeres are epigenetically marked by specialized nucleosomes and incorporate specific histone variants (CenH3) (Stoler *et al.* 1995; Keith and Fitzgerald-Hayes 2000; Bernad *et al.* 2009). CENP-A (histone H3-like centromeric protein A) in humans, CID in flies, and Cse4 (chromosome segregation protein 4) in budding yeast contribute to the formation of specialized nucleosomes making up the centromere, the chromosome attachment site to the mitotic spindle, and set the foundation for the hierarchical assembly of the kinetochore (Meluh and Koshland 1997). Following the first reports that *Saccharomyces cerevisiae* possesses a single nucleosome structure (Meluh and Koshland 1997; Furuyama and Biggins 2007), more recent findings suggest that there are additional Cse4 molecules and 0.5/3 variant nucleosomes at the centromere (Maresca 2013). Histone variant mislocalization or its overexpression may create putative sites of erroneous attachment of the chromosomes to the mitotic spindle, thus inducing aberrant segregation and aneuploidy (Tomonaga *et al.* 2003; Heun *et al.* 2006; Moreno-Moreno *et al.* 2006). In yeast, the centromeric histone variant Cse4 marks the centromere and regulates the behavior and segregation of chromosomes in mitosis. Mislocalization of centromeric histone causes aberrant attachment sites and mitotic instability. Proteasomal degradation and removal of CenH3 from ectopic localization is therefore a fundamental regulatory step, achieved through the deposition of a poly-Ub chain on Cse4 by Psh1 (Collins *et al.* 2007; Hewawasam *et al.* 2010; Ranjitkar *et al.* 2010). This process ensures the exclusive localization of Cse4 at the centromere (Folco and Desai 2010). Surprisingly, Psh1 deletion does not induce mitotic defects in yeast, implying that additional mechanisms might be involved (Folco and Desai 2010). Since ubiquitylation plays a crucial role in Cse4 proteolysis, we reasoned that a counteracting deubiquitylation activity could also be important. Here we demonstrate, at the genetic level, that Psh1 is epistatic to the Ub-protease Ubp8 and that its absence causes a high rate of mitotic instability and minichromosome loss rescued by further deletion of Psh1. Additionally, we show that Ubp8 directly deubiquitylates Cse4 and that its deletion produces not only the accumulation of a short Ub-oligomer on the centromeric histone but also its faster proteolytic degradation. Finally, our results suggest that the presence of this short ubiquitin mark on Cse4 counteracts the elongation of polyubiquitin chains by Psh1 and represents a poor mark for removal from ectopic localization. In brief, we describe a new function for the SAGA complex and the DUB-Ubp8 component. We propose a model in which Ubp8 is directly involved in the regulation of the ubiquitylation status of the histone variant Cse4, and we suggest that Ubp8 contributes not only to the mechanism ensuring its restricted localization at the centromere but also to the general mitotic stability of the cell.

## Materials and Methods

### Materials, yeast strains and culture

All media components were either from BD or GIBCO, general reagents from Sigma Aldrich and Roche unless otherwise stated. Yeast strains were from a w303 isogenic background. All strains are listed in Table 1. Gene disruption and protein tagging were performed as previously described (Janke *et al.* 2004) and controlled by PCR amplification and western blot assays. Growth media were: YPD (1% yeast extract, 2% bactopeptone, 2% glucose), SD (0.67% YNB and 2% glucose) and SC (0.67% YNB, 2% glucose or galactose, selective Drop-Out Mix). Benomyl was used at a final concentration of 10/15μγρ/μl. Antibodies were: anti-Myc (clone 9E10, sc-40, Santa Cruz), anti-ADA2 (sc-6651, Santa Cruz), anti-HA (sc-7392, Santa Cruz), and Anti-6X His tag (ab1187, Abcam).

**Table 1:** Yeast strains used in Canzonetta *et al.*

Name	Genotype	Source
W303	*MAT****a*** *ade2-1 trp1-1 leu2-3*, *112 his3-11,15 ura3 can1-100 ssd1.*	(Vernarecci *et al.* 2008)
yFT21	*MAT****a*** *ade2-1 trp1-1 leu2-3*, *112 his3-11,15 ura3 can1-100* ssd1. *ubp8*::*HIS3MX6*	This work
yCB12	*MAT****a*** *ade2-1 trp1-1 leu2-3*, *112 his3-11,15 ura3 can1-100* ssd1. *psh1*::*HIS3MX6*	This work
ySP1090	*MATα*, *cse4-1 ade2-101 his3D-100 leu2-3 LYS2 trp1D*, *ura3-52.*	(Stoler *et al.* 1995)
yCC11	*MATα*, *cse4-1 ade2-101 his3D-100 leu2-3 LYS2 trp1D*, *ura3-52. ubp8*::*HIS3MX6.*	This work
yCC12	*MATα*, *cse4-1 ade2-101 his3D-100 leu2-3 LYS2 trp1D*, *ura3-52. psh1*::*HIS3MX6.*	This work
CBY3	*MAT****a*** *ade2-1 trp1-1 leu2-3*, *112 his3-11,15 ura3 can1-100 ssd1*. pRS415-ADE2 1225 (CEN/ARS LEU2).	This work
CBY8	*MAT****a*** *ade2-1 trp1-1 leu2-3*, *112 his3-11,15 ura3 can1-100 ssd1*. pRS415-ADE2 1225 (CEN/ARS LEU2). *ubp8*::*HIS3MX6*.	This work
CBY13	*MAT****a*** *ade2-1 trp1-1 leu2-3*, *112 his3-11,15 ura3 can1-100 ssd1*. pRS415-ADE2 1225 (CEN/ARS LEU2). *psh1*::*HIS3MX6*.	This work
CMY13	*MAT****a*** *ade2-1 trp1-1 leu2-3*, *112 his3-11,15 ura3 can1-100 ssd1*. pRS415-ADE2 1225 (CEN/ARS LEU2). *ubp8*::*HIS3MX6. psh1*::*KanMX6*	This work
CBY4	*MAT****a*** *ade2-1 trp1-1 leu2-3*, *112 his3-11,15 ura3 can1-100 ssd1*. pRS415-ADE2 1225 (CEN/ARS LEU2). *gcn5*::*KanMX6*.	This work
CBY9	*MAT****a*** *ade2-1 trp1-1 leu2-3*, *112 his3-11,15 ura3 can1-100 ssd1*. pRS415-ADE2 1225 (CEN/ARS LEU2). *ubp8*::*HIS3MX6. gcn5*::*KanMX6*.	This work
CBY15	*MAT****a*** *ade2-1 trp1-1 leu2-3*, *112 his3-11,15 ura3 can1-100 ssd1*. pRS415-ADE2 1225 (CEN/ARS LEU2). *ada3*::*HIS3MX6*.	This work
CBY21	*MAT****a*** *ade2-1 trp1-1 leu2-3*, *112 his3-11,15 ura3 can1-100 ssd1*. pRS415-ADE2 1225 (CEN/ARS LEU2). *Sgf73*::*HIS3MX6*.	This work
SBY3570	*MAT****a*** *ura3-1 leu2,3-112 his3-11 trp1-1 ade2-1 can1-100 (pSB816*, *pGAL-MYC13-CSE4*, *URA3*, *2μ)*	(Collins *et al.* 2004)
SBY3571	*MAT****a*** *ura3-1 leu2,3-112 his3-11 trp1-1 ade2-1 can1-100 (pSB817*, *pGAL-MYC13-CSE4-16R*, *URA3*, *2μ)*	(Collins *et al.* 2004)
FTY22	*MAT****a*** *ura3-1 leu2,3-112 his3-11 trp1-1 ade2-1 can1-100 (pSB816*, *pGAL-MYC13-CSE4*, *URA3*, *2μ). ubp8*::*HIS3MX6.*	This work
RBY013	*MAT****a*** *ura3-1 leu2,3-112 his3-11 trp1-1 ade2-1 can1-100 (pSB817*, *pGAL-MYC13-CSE4-16R*, *URA3*, *2μ). ubp8*::*HIS3MX6.*	This work
CBY17	*MAT****a*** *ura3-1 leu2,3-112 his3-11 trp1-1 ade2-1 can1-100 (pSB816*, *pGAL-MYC13-CSE4*, *URA3*, *2μ). psh1*::*HIS3MX6.*	This work
CMY6	*MAT****a*** *ura3-1 leu2,3-112 his3-11 trp1-1 ade2-1 can1-100 (pSB817*, *pGAL-MYC13-CSE4-16R*, *URA3*, *2μ). psh1*::*HIS3MX6.*	This work
CBY24	*MAT****a*** *ura3-1 leu2,3-112 his3-11 trp1-1 ade2-1 can1-100 (pSB816*, *pGAL-MYC13-CSE4*, *URA3*, *2μ). ubp8*::*HIS3MX6. psh1*::*KanMX6*	This work
yML48	*MAT****a*** *ura3-1 leu2,3-112 his3-11 trp1-1 ade2-1 can1-100 (pSB817*, *pGAL-MYC13-CSE4*, *URA3*, *2μ). ubp8*::*HIS3MX6. psh1*::*KanMX6*	This work
CMY19	*MAT****a*** *ura3-1 leu2,3-112 his3-11 trp1-1 ade2-1 can1-100 (pSB816*, *pGAL-MYC13-CSE4*, *URA3*, *2μ). Ubp10*::*HIS3MX6.*	This work
CMY20	*MAT****a*** *ura3-1 leu2,3-112 his3-11 trp1-1 ade2-1 can1-100 (pSB817*, *pGAL-MYC13-CSE4-16R*, *URA3*, *2μ). Ubp10*::*HIS3MX6.*	This work
SBY617	*MAT****a*** *ade2-1 ura3-1 leu2-3,112 his3-11 trp1-1 can1-100 bar1*Δ *CSE4-MYC12*::*URA3.*	(Buvelot *et al.* 2003)
yFT17	*MAT****a*** *ade2-1 ura3-1 leu2-3,112 his3-11 trp1-1 can1-100 bar1*Δ *CSE4-MYC12*::*URA3 ubp8*::*HIS3MX6.*	This work
yCB16	*MAT****a*** *ade2-1 ura3-1 leu2-3,112 his3-11 trp1-1 can1-100 bar1*Δ *CSE4-MYC12*::*URA3 psh1*::*HIS3MX6.*	This work
yCC1	*MAT****a*** *ade2-1 ura3-1 leu2-3,112 his3-11 trp1-1 can1-100 bar1*Δ *CSE4-MYC12*::*URA3 ubp8*::*HIS3MX6*, *psh1*::*KanMX6*.	This work
yCB21	*MAT****a*** *ade2-1 trp1-1 leu2-3*, *112 his3-11,15 ura3 can1-100 ssd1. (pJD421 His-Ubiquitin*::*LEU).*	This work
yCM2	*MAT****a*** *ade2-1 trp1-1 leu2-3*, *112 his3-11,15 ura3 can1-100 ssd1. CSE4-MYC12*::*URA3. (pJD421 His-Ubiquitin*::*LEU).*	This work
yCM4	*MAT****a*** *ade2-1 ura3-1 leu2-3,112 his3-11 trp1-1 can1-100 bar1*Δ *CSE4-MYC12*::*URA3 ubp8*::*HIS3MX6. (pJD421 His-Ubiquitin*::*LEU)*	This work
YCC9	*MAT****a*** *ade2-1 ura3-1 leu2-3,112 his3-11 trp1-1 can1-100 bar1*Δ *CSE4-MYC12*::*URA3*, *psh1*::*HIS3MX6*, *(pJD421 His-Ubiquitin*::*LEU)*	This work
YCC10	*MAT****a*** *ade2-1 ura3-1 leu2-3,112 his3-11 trp1-1 can1-100 bar1*Δ *CSE4-MYC12*::*URA3*, *ubp8*::*HIS3MX6*, *psh1*::*KanMX6*, *(pJD421 His-Ubiquitin*::*LEU)*	This work
yCC15	*MAT****a*** *ade2-1 trp1-1 leu2-3*, *112 his3-11,15 ura3 can1-100 ssd1. CSE4-MYC12*::*URA3*, *doa1*::*KanMX6*, *(pJD421 His-Ubiquitin*::*LEU)*.	This work
yCC14	*MAT****a*** *ade2-1 trp1-1 leu2-3*, *112 his3-11,15 ura3 can1-100 ssd1. CSE4-MYC12*::*URA3*, *ubp8*::*HIS3MX6*, *doa1*::*KanMX6*, *(pJD421 His-Ubiquitin*::*LEU)*.	This work
yFT33	*MAT****a*** *ade2-1 trp1-1 leu2-3*, *112 his3-11,15 ura3 can1-100 ssd1. Ubp8-MYC9*::*HIS3MX6*	This work
yCC21	*MAT****a*** *ade2-1 trp1-1 leu2-3*, *112 his3-11,15 ura3 can1-100 ssd1. Ubp8-MYC9*::*HIS3MX6*, *CSE4-HA6*:: klTrp1	This work

### Color sectoring

WT (wild-type, CBY3), *psh1*Δ (CBY13), *ubp8*Δ (CBY8), *ubp8*Δ*psh1*Δ (CMY13), *gcn5*Δ (CBY4), *gcn5*Δ*ubp8*Δ (CBY9), *ada3*Δ (CBY24) *and sgf73*Δ (CBY21) strains containing the centromeric plasmid pRS415-1225 (CEN/ARS LEU2/ADE2) were grown to early log phase in selective ade-leu- minimal medium at 28°. Cells were then diluted to 2 × 10^3^ cells/ml, plated on YPD plates, and grown for 3–4 days. Colonies were then scored as white (pRS415-1225 retention) or red (pRS415-1225 loss) or sectorialized (pRS415-1225 partial loss) (Ma *et al.* 2010).

### Cycloheximide degradation assay

Cells expressing tagged proteins (Cse4-Myc) were grown in 100 ml of YPD to an OD_600_ of 0.4. A 20 ml aliquot was collected before adding cycloheximide to a final concentration of 10 μg/ml (time zero). Subsequent shares were harvested every 40 min up to 2 hr and processed promptly accordingly to Knop *et al.* (1999) with minor modifications. Briefly, cells were spun down at 3500 rpm for 5 min, resuspended in 0.3 M NaOH, 140 mM BME, vortexed, and incubated on ice for 15 min with occasional vortexing. TCA (55%) was added to a final concentration of 6.5% followed by further incubation on ice. Samples were centrifuged at 4° at maximum speed for 20 min. Protein pellets were resuspended in HU buffer (8 M urea, 5% SDS, 200 mM Tris pH 6.8, 0.1 mM EDTA, 100 mM DTT, bromophenol blue), and loaded on 10% SDS-PAGE gels. Western blots were hybridized with anti-myc, and anti-Ada2 signal was used as a loading control.

### Coimmunoprecipitation

Yeast strains were grown overnight in the appropriate medium, diluted to an OD_600_ of 0.25 in YPD, and collected to an OD_600_ of 0.6; 100 ml of the exponentially growing cells were then harvested and incubated in spheroplast buffer [1.2 M sorbitol, 50 mM potassium phosphate (pH 7.4), 1 mM MgCl, 250 μg of zymolyase per ml], with shaking for 1 hr at 30°. Spheroplasts were harvested by centrifugation for 2 min at 5000 rpm, washed with 1.2 M sorbitol, and pelleted at low speed as before. All subsequent steps were performed at 4°. Spheroplasts were lysed for 15 min on ice in IP buffer [50 mM Tris (pH 8), 1 mM EDTA, 50 mM NaCl, 1% NP-40] plus protease inhibitors. Lysates were centrifuged at 13,000 rpm for 5 min and the pellet was discarded. Supernatants were precleared by incubation for 1 hr with protein G magnetic beads; 1/20^th^ of the crude extract was kept as input material. Immunoprecipitation was performed overnight at 4° on a rotating wheel with 10 µl of G-conjugated Dynabeads (Invitrogen) that had been prebound with an anti-myc antibody. Beads were collected in the morning, washed extensively with IP buffer, and resuspended in 30 µl HU-buffer. Proteins were released from the beads after 10 min incubation at 65°. Supernatant was either used immediately or frozen at −80°. Eluates and Inputs were loaded on 7.5% and 10% SDS-PAGE gels, respectively. Western blots were hybridized with anti-myc, Anti-HA, and anti-Ada2 antibodies. Signals were detected by chemiluminescence using LiteAblot Extend (EuroClone) and the ChemiDoc XRS+ System (Biorad).

### Purification of ubiquitylated proteins

Cells transformed with a plasmid encoding 6His-ubiquitin under the CUP1 promoter [YEp96-6His-Ub, (Iglesias *et al.* 2010)] were grown on selective media and stimulated overnight with 0.1 mM CuSO_4_. Growth was stopped in the morning at an OD_600_ of 0.8–1. Cells were lysed in guanidinium buffer (6 M guanidinium-HCl, 0.1 M Na_2_HPO_4_/NaH_2_PO_4_, 0.01 M Tris-HCl, pH 8.0, 0.1% Triton X-100, 5 mM imidazole, 10 mM β-mercaptoethanol, 0.1 mM MG132 and protease inhibitors), 1/20^th^ of the lysate was kept aside as input material and TCA (5.5%) precipitated; the rest was incubated on a Ni-NTA agarose resin (QIAGEN) at 4° with rotation. The resin was washed three times with urea buffer (8 M urea, 0.1 M Na_2_HPO_4_/Na_2_HPO_4_, 0.01 M Tris-HCl, pH 6.4, 3.5 mM β-mercaptoethanol, and 0.1% Triton X-100) before elution in 2X sample buffer (0.125 M Tris-HCl, pH 6.8, 4% SDS, 0.285 M β-mercaptoethanol, 20% SDS, 2% bromophenol blue). Eluates and inputs were loaded on 7.5% and 10% SDS-PAGE gels, respectively. Western blots were hybridized with anti-myc, Anti-6X His tag, and anti-Ada2 antibodies. Signals were detected by chemiluminescence using LiteAblot Extend (EuroClone) and the ChemiDoc XRS+ System (Biorad).

### Chromatin immunoprecipitation

ChIP was performed as described (Vernarecci *et al.* 2008): 100 ml of an exponentially growing culture (OD_600_ 0.3–0.6) were fixed for 60 min with 1% formaldehyde, then harvested and washed twice in 20 ml of Tris-buffered saline (20 mM Tris-HCl, pH 7.5, 150 mM NaCl). Lysis was performed in 500 μl ice cold lysis buffer (1 mM EDTA, 50 mM HEPES, 140 mM NaCl, 1% Triton X-100, 1 mg/ml Na deoxycholate) with glass beads by vortexing 10 times for 30 sec at 4°. Chromatin was collected and shared by sonication (four times, 30 sec ON/30 sec OFF on ice) using a Branson Digital Sonifier (Branson Ultrasonic Corporation, Danbury, CT) on 10% impulse (average fragment size 500 bp) then clarified for 5 min in a microcentrifuge. For immunoprecipitation, solubilized chromatin was incubated overnight at 4° on a rotating wheel with 10 µl of G-conjugated Dynabeads (Invitrogen) that had been prebound with anti-myc or anti-HA antibodies. Immunoprecipitates were collected using a step-wise washing protocol. The immunocomplexes were eluted by adding 0.25 ml Elution buffer (50 mM Tris-Cl, pH 7.5, 10 mM EDTA, 1% SDS), and incubated at 65° for 10 min. Eluates were phenol chloroform and ethanol precipitated. Immunoprecipitated DNA was analyzed by real-time qPCR (Bioline, Sensimix, QT615-02). Real-time qPCR was performed using a BioRad iCycler iQ (BioRad).

#### Primer sequences:

CEN3F 5′-gatcagcgccaaacaatatgg-3′, CEN3R 5′-aacttccaccagtaaacgtttc-3′
TEL6F-5′ccataatgcctcctatatttagccttt-3′, TEL6 R 5′-tgacatatccttcacgaatattgttaga-3′
NTSF-5′atgttcagtaggtgggagtgagag-3′, NTSR-5′catcccggtgccgtaaatgcaaaac-3′

### Data analysis and statistical analyses

Each experiment represents the average of at least three different biological replicates unless otherwise stated in the figure legend. Standard errors are shown and asterisks are as follows: ** *P* < 0.01, * < 0.05. ChIP experiment analysis: equal volumes from input and immunoprecipitated samples were subjected to real-time qPCR. Reactions were carried out in duplicate in 25 μl volumes. The enrichment of a given target sequence was determined as the fold difference between the amount of target sequence in the immunoprecipitated fraction and the amount of target sequence in the input DNA (IP/Input). In each ChIP experiment, fold difference values obtained for the WT strain, with each primer pair, were used for standardization. For this reason, the WT values are set to one and do not present error bars. Average fold enrichment was thus calculated for *ubp8*Δ and *psh1*Δ strains and error bars represent the standard errors of the set of values obtained for each PCR amplicon position. Background immunoprecipitation with nonspecific antibody (anti-HA) and no antibody was also determined for each experiment and subtracted to the specific signal for each primer pair. Cycloheximide protein analysis: signals obtained for Cse4-myc and ADA2 were quantified with Optiquant densitometry software. Each value obtained for Cse4-myc at a specific time point was normalized to its equivalent ADA2 signal. For each yeast strain tested, the protein levels were referred to the respective time zero (no cycloheximide).

### Data availability

Strains are available upon request.

## Results

### Mitotic stability is compromised by loss of Ubp8

**Figure 1:**
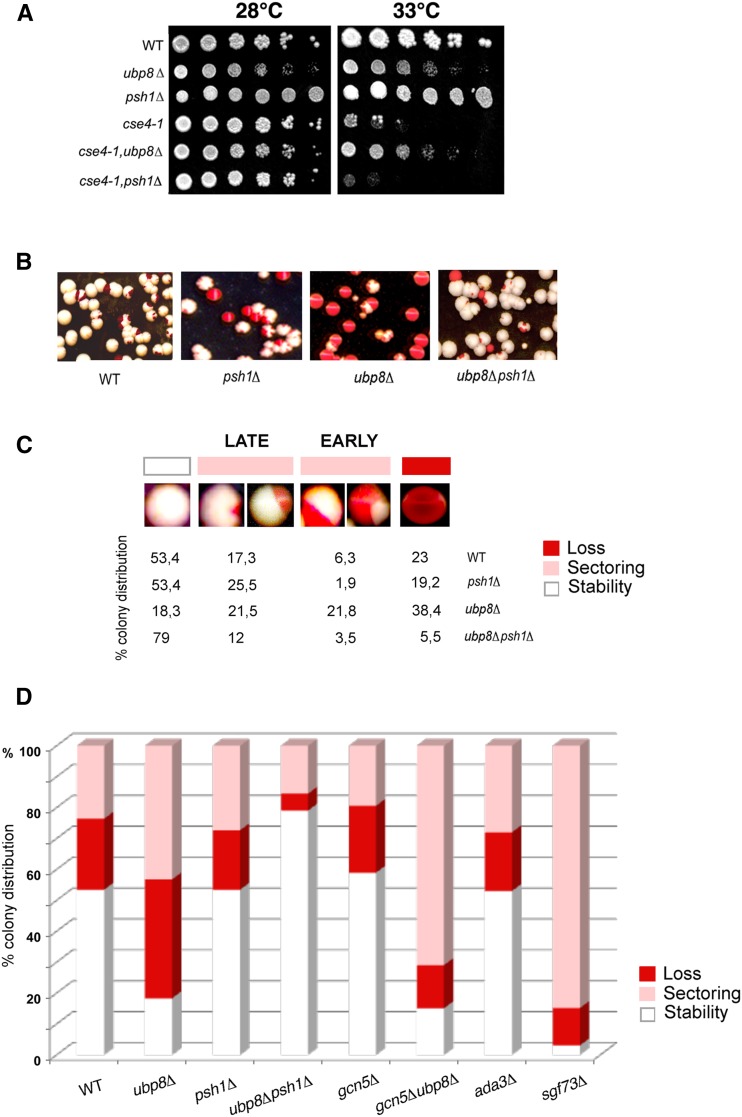
Ubp8 interacts with CEN-H3 TS-allele *cse4-1* at the genetic level and its loss increases mitotic stability. (A) Comparative growth spot assay of fivefold serial dilutions of WT, *ubp8*Δ; *psh1*Δ; *cse4-1*; *cse4-1*, *ubp8*Δ; *cse4-1*, *psh1*Δ strains plated on YPD and grown at permissive (28°) and nonpermissive (33°) temperatures for 2 days. Strain with TS-allele *cse4-1* was assayed in the absence of DUB-Ubp8 (*cse4-1*, *ubp8*Δ) and E3-Ub ligase Psh1 (*cse4-1*, *psh1*Δ). (B) WT, *ubp8*Δ, *psh1*Δ and *ubp8*Δ*psh1*Δ strains carrying the stable ADE/LEU pRS425-1225 CEN/ARS plasmid were grown in selective medium (leu), plated on YPD, grown, and stored at 4° for red color accumulation. (C) Cell percentage (%) showing stable, sectorialized (late and early minichromosome loss), and unstable mitotic phenotype in WT, *ubp8*Δ, *psh1*Δ and *ubp8*Δ*psh1*Δ. (D) Plasmid loss rates of WT, *psh1*Δ, *ubp8*Δ, *ubp8*Δ*psh1*Δ, *gcn5*Δ, *gcn5*Δ*ubp8*Δ, *sgf73*Δ and *ada3*Δ strains. Plasmid loss is expressed as the relative percentage of white, red, and sectorialized colonies grown on plates (400-600 colonies counted for each strain). WT, wild-type; YPD, yeast extract peptone dextrose.

It has been shown that the SAGA DUB subunit Ubp8 deubiquitylates histone H2B (Ingvarsdottir *et al.* 2005). We wanted to investigate whether Ubp8 might also be functionally active on the centromeric histone variant Cse4. We first assayed, at the genetic level, putative interactions between a mutant carrying the TS-allele *cse4-1* and deletion of the DUB Ub-protease Ubp8 and the E3-Ub ligase Psh1, respectively. The growth spot assay in Figure 1A clearly shows that deletion of Ubp8 at a nonpermissive temperature (33°) rescues the growth defect of the TS-allele *cse4-1*, as shown in the *cse4-1,ubp8*Δ mutant, suggesting a genetic interaction between Ubp8 and Cse4. Psh1 deletion at a nonpermissive temperature appeared to be synthetically lethal in the *cse4-1*,*psh1*Δ mutant, confirming a genetic correlation between Psh1 and Cse4. Lack of Ubp8 rescues the temperature sensitivity of the *cse4-1* mutant. Starting from this evidence, we followed several experimental lines in order to demonstrate the genetic interaction found. We examined whether lack of Ubp8 might affect mitotic stability. Using a color-sectoring assay, we were able to visualize the segregation of a stably inherited centromeric plasmid (Zhang *et al.* 2008). The CEN plasmid was intentionally chosen in order to provide an assay to measure the behavior of the centromere, more than an absolute mitotic stability value. Strains deleted in different SAGA components were grown on YPD complete solid medium, cells with defects in plasmid maintenance and transmission appeared red or partially sectorialized (white/red), while those stably segregating appeared white. WT, *psh1*Δ, *ubp8*Δ and *ubp8*Δ*psh1*Δ strains are shown in Figure 1B. The percentage of stably segregating white cells was calculated for each strain and compared to the sectorialized and red colonies (Figure 1C). The fraction of white stably segregating colonies was very similar between the WT and *psh1*Δ strains, confirming the absence of mitotic defects in the Psh1-deleted mutant (Hewawasam *et al.* 2010). Surprisingly, the *ubp8*Δ strain showed a drastic increase in plasmid loss, with most of the colonies being sectorialized or red, clearly indicating a significant increase in centromere instability. Interestingly, the double *ubp8*Δ*psh1*Δ strain (Figure 1, B–D) completely rescued loss of the CEN plasmid found in the *ubp8*Δ strain, thus excluding additional indirect effects due to Ubp8 deletion and indicating an epistatic link between Psh1 and Ubp8. Strains deleted in other SAGA components were similarly analyzed. Deletion of the HAT Gcn5 alone did not produce mitotic defects while CEN plasmid loss was drastically increased in the double *gcn5*Δ*ubp8*Δ strain (Figure 1D), underlying once more the effect of Ubp8. We also tested the deletion effects of Sgf73, and of the transcriptional adaptor Ada3 (Figure 1D). The instability observed in the *sgf73*Δ strain is again linked to Ubp8, since this subunit is required for correct incorporation of Ubp8 in the DUB module (Samara *et al.* 2010). Lack of Ada3, instead, had no effect, ruling out other putative SAGA-dependent transcriptional defects. This analysis suggests that the centromere instability defect can be specifically attributed to absence of the DUB-Ubp8 deubiquitylase. Collectively, our data show that the histone variant Cse4 interacts at the genetic level with Psh1 and DUB-Ubp8, and that deletion of Ubp8 significantly increases the loss of a stably inherited minichromosome.

### Ubp8 interacts with Cse4 at the physical level

**Figure 2:**
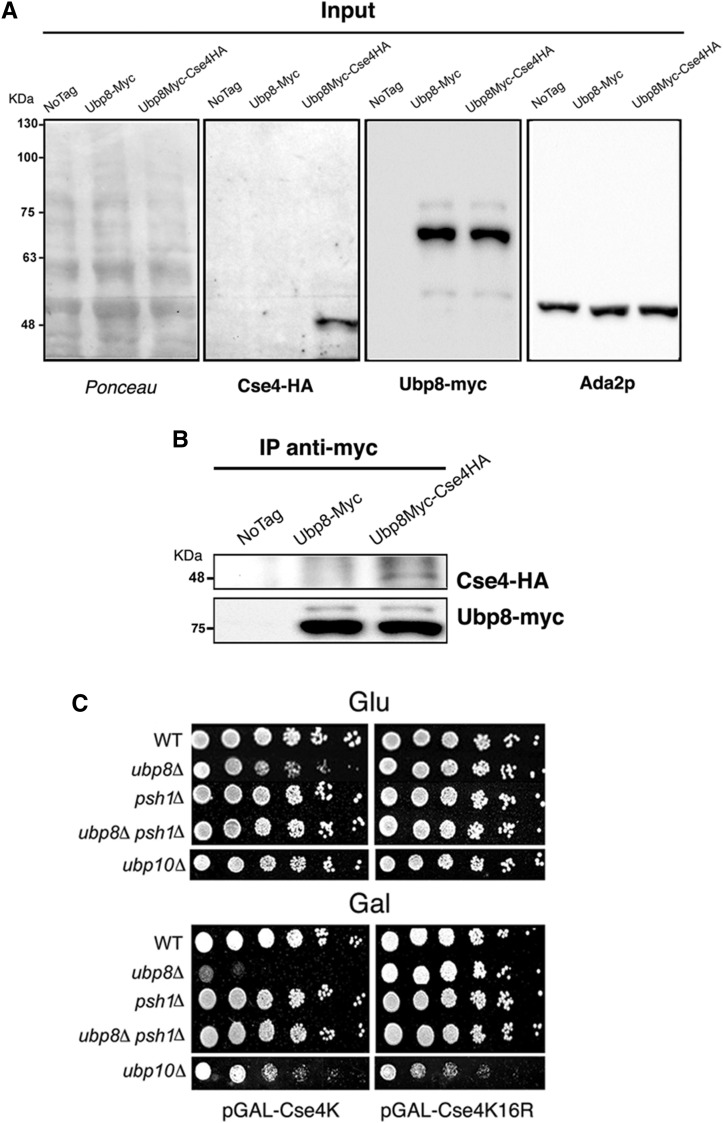
Ubp8 interacts with Cse4 at a physical level and acts on Cse4 lysines. (A) Inputs from WT (wild-type), Ubp8-myc, and Ubp8myc-Cse4HA strains were run on 10% PAGE, transferred on nitrocellulose membrane, stained with red-ponceau, and consecutively probed with anti-HA (Cse4-HA), anti-myc (Ubp8-myc), and anti-ADA2 (internal protein control) antibodies. (B) Coimmunoprecipitation (coIP) carried out on whole cell extracts shown in (A) indicates that Ubp8-myc is able to coIP Cse4-HA in the double-tagged strain. (C) Growth spot assay. Fivefold serial dilutions of WT, *ubp8*Δ, *psh1*Δ, *ubp8*Δ-*psh1*Δ, and *ubp10*Δ strains expressing pGAL-Cse4 or pGAL-Cse4^K16R^ were plated in glucose (GLU) or inducing galactose (GAL) SC-media and grown for 5 d at 28°.

Building on these genetic results, we wanted to exclude off-target effects and determine whether a direct physical interaction existed between Ubp8 and Cse4. The DUB module is composed of the Sgf73 (SaGa associated factor, 73 kDa), Sgf11 (SaGa associated factor 11 kDa), Sus1 (Sl gene upstream of ySa1), and Ubp8 subunits. Sgf73 is required for the incorporation of Ubp8 in the complex and interacts with the structural subunit Spt7 (Han *et al.* 2014). It has been reported that Spt7 itself interacts with Cse4 (Ranjitkar *et al.* 2010), suggesting that a direct interaction between the SAGA complex and Cse4 exists. To provide evidence of a physical interaction between Ubp8 and Cse4, coimmunoprecipitation (coIP) was performed on a double epitope-tagged strain carrying both Cse4-6HA and Ubp8-9myc. A single Ubp8-9myc-tagged strain was used as a control. Correct expression of tagged proteins was verified by western blot analysis of total extracts (Figure 2A). Cse4 is not an abundant protein and the tagged Cse4-HA assayed is expressed at physiological levels. Nevertheless, when immunoprecipitation of Ubp8-myc was performed, using an anti-myc antibody, a specific signal corresponding to Cse4-6HA (48 kDa) was observed in the double-tagged strain (Figure 2B) but not in the two negative controls (the untagged and the single Ubp8-9myc strains). Ubp8-myc (75 kDa) was successively detected in both immunoprecipitated strains, confirming that anti-myc immunoprecipitation was successful. Taken together, these results demonstrate that Ubp8 interacts with Cse4 at the genetic and physical levels.

### Ubp8 acts on Cse4 lysines, and absence of E3-ubiquitin ligase Psh1 rescues Ubp8 deletion

Our results indicate that Ubp8 genetically interacts with Cse4 and that its absence enhances mitotic instability. Since Cse4 is ubiquitylated and Ub-dependent proteolysis regulates Cse4 localization, we speculated that Ubp8 could be directly engaged in targeting Cse4 lysines. We used a strain overexpressing a WT copy of Cse4 under the control of a Gal promoter (pGAL-Cse4) or a lysine-free version carrying substituted arginines (pGAL-Cse4^K16R^) that prevents ubiquitylation (Au *et al.* 2008; Mena *et al.* 2009). pGAL-Cse4 and pGAL-Cse4^K16R^ were tested in strains lacking the ubiquitin proteases *ubp8*Δ and *ubp10*Δ or the E3-Ub ligase *psh1*Δ. The results are shown in the growth spot assay under noninducing (glucose) or inducing (galactose) conditions (Figure 2C). Overexpression of pGAL-Cse4 was clearly synthetically lethal in *ubp8*Δ but completely ineffective if the unmodifiable version pGAL-Cse4^K16R^ was expressed. Interestingly, the double-deleted strain *ubp8*Δ*psh1*Δ expressing pGAL-Cse4 showed a growth rescue comparable to that obtained by expressing the *pGAL*-Cse4^K16R^ version in the *ubp8*Δ strain. Lack of growth defects in the Cse4^K16R^-substituted mutant or in absence of the Ub-ligase Psh1 clearly indicate a selective role for Ubp8 on Cse4 lysines. It was reported that histone H2B-K123Ub is deubiquitylated by both DUBs, Ubp8 and Ubp10 (Schulze *et al.* 2011). To assay a putative role of Ubp10 on Cse4, both pGAL-Cse4 and pGAL-Cse4^K16R^ were overexpressed in the *ubp10*Δ strain. No effect was observed, indicating that Ubp8 and not Ubp10 targets Cse4. Collectively, these results indicate that Ubp8 is acting on Cse4 lysines, since its deletion is ineffective when these are substituted by unmodifiable arginines (Cse4^K16R^) or when Ub-ligase Psh1 is eliminated and ubiquitin is not deposed. These data reinforce the epistasis and functional interaction between Psh1 and Ubp8, suggesting a role for ubiquitin protease Ubp8 in Cse4 deubiquitylation.

### Ubiquitin mediated proteolysis of Cse4 is enhanced in the absence of Ubp8

**Figure 3:**
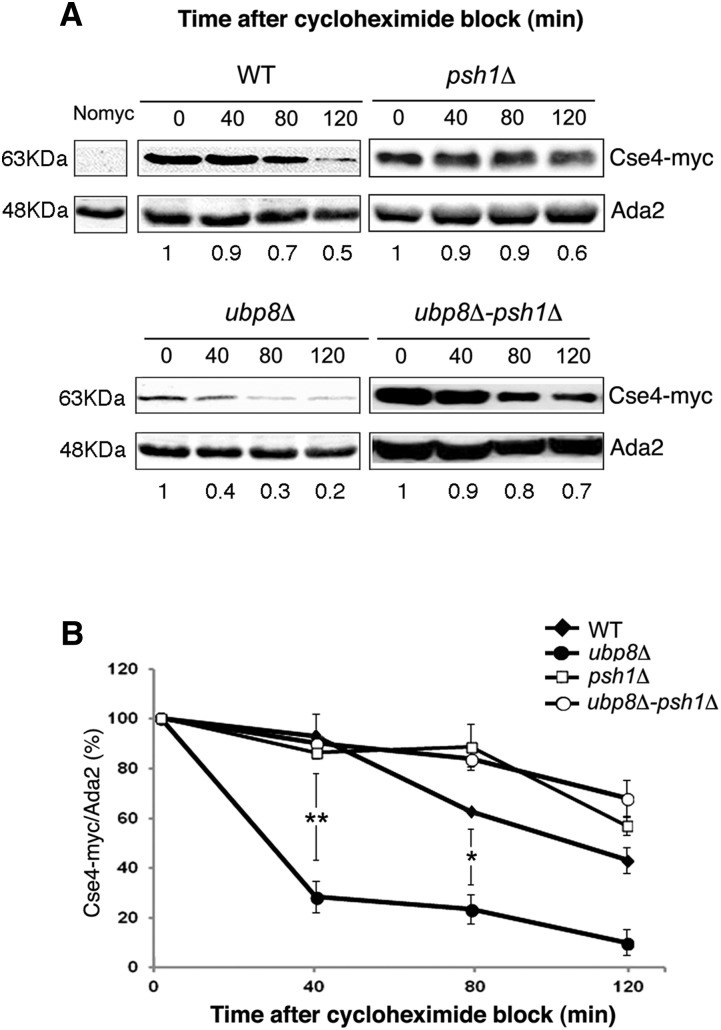
Lack of Ubp8 causes faster proteolysis of Cse4-myc rescued by concomitant deletion of the E3-Ub ligase Psh1. (A) Proteins were extracted after adding cycloheximide (CHX) (10 μg/ml) from WT (wild-type), *ubp8*Δ, *psh1*Δ, and *ubp8*Δ*psh1*Δ strains containing an integrated copy of Cse4-myc. Lysates prepared at indicated time points (min) were probed with anti-myc and anti-ADA2 antibodies. The relative amount of Cse4-myc at each time point was quantified and normalized to Ada2 levels as indicated under each lane. ADA2-normalized Cse4 protein levels at each time point of CHX treatment are presented as a fraction of the remaining amount relative to the sample t = 0. (B) The graph shows the percentage of Cse4-myc signal normalized to Ada2 in each strain at the indicated time points. WT closed diamonds, *ubp8*Δ closed circles, *psh1*Δ open squares, *ubp8*Δ-*psh1*Δ open circles. Standard errors of the means are shown. Asterisks are as follows: ** *P* < 0.01, * < 0.05.

CenH3 Cse4 is an unstable protein whose ubiquitin-mediated proteolysis contributes to its exclusive localization at the centromere (Collins *et al.* 2004). Since our data provided evidence for both genetic and physical interactions between Ubp8 and Cse4, we reasoned that DUB Ubp8 might play a direct role in Cse4 deubiquitylation and thus interfere with the protein degradation rate. Cse4-myc steady-state levels were assessed upon *de novo* protein translation block with cycloheximide in WT, *ubp8*Δ, *psh1*Δ, and *ubp8*Δ*psh1*Δ strains (Figure 3A). Interestingly, in the absence of Ubp8, Cse4-myc levels were drastically reduced after 40 min of treatment (Figure 3A). Previous reports have shown that proteolytic degradation depends on lysine ubiquitylation (Ranjitkar *et al.* 2010); similarly, we noted that Cse4p is stabilized in *psh1*Δ cells (Figure 3A). Based on these observations, we expected that a subsequent deletion of the E3-Ub ligase Psh1 in the *ubp8*Δ strain would reverse Cse4p faster proteolysis. Consistent with this assumption, Cse4p was highly stabilized and its degradation significantly reduced in the *ubp8*Δ*psh1*Δ strains. Band intensities were quantified and the results summarized in the histograms in Figure 3B. The y axis reports the relative amount of Cse4-myc/ADA2 at each point referred to the 100% value at time 0 for each strain. This observation, our genetic data and coimmunoprecipitation experiments all suggested a direct link between DUB-Ubp8 and the centromeric histone Cse4. Moreover, the strong recovery in protein stability observed in the double-deleted *ubp8*Δ*psh1*Δ strain clearly indicated a role for Ubp8 in removing ubiquitin from Cse4 lysines downstream of Psh1. These results prompted us to investigate the status of Cse4 ubiquitylation at the biochemical level in order to directly demonstrate this novel activity of the Ub-protease Ubp8.

### Ubp8 deubiquitylates Cse4 and removes a short Ub-oligomer

**Figure 4:**
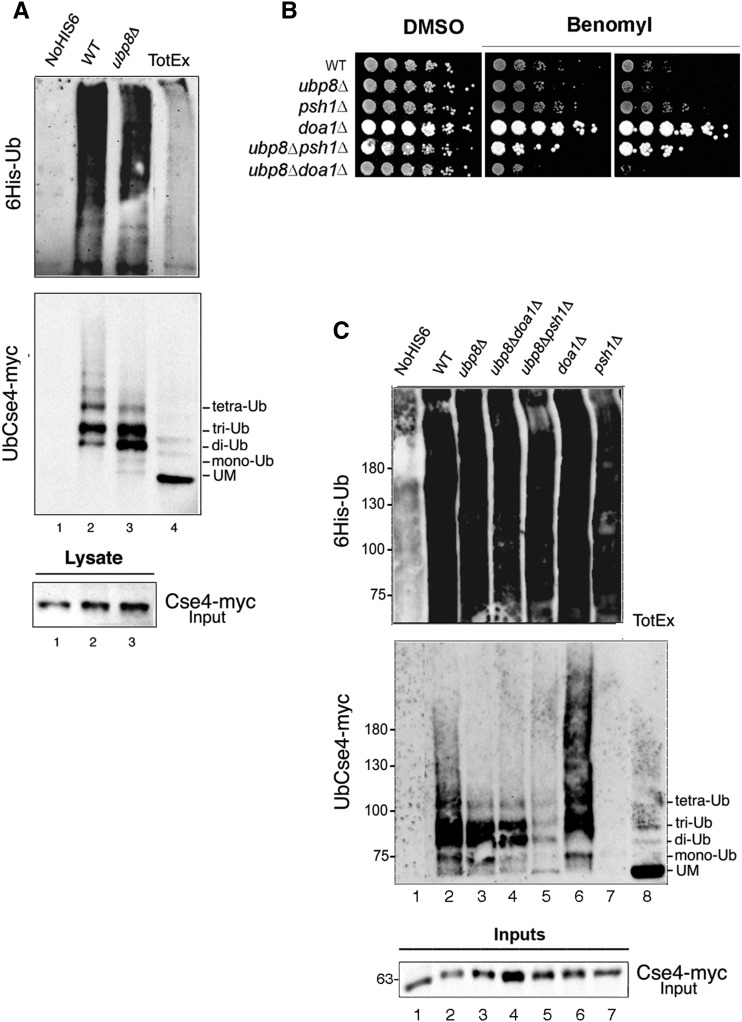
SAGA-DUB Ubp8 removes a short ubiquitin oligomer from the centromeric histone Cse4. (A) Cse4-myc tagged strains containing no vector, lane 1, or a PCUP1:6HIS-Ubiquitin plasmid (WT, 2; *ubp8*Δ, 3). 6HIS-Ub conjugates purified on a Ni+ column were run in parallel with a wild-type (WT) total extract, lane 4. Western blot was sequentially hybridized with anti-myc and anti-6His antibodies; 1/20^th^ of total extracts were run separately and hybridized with anti-myc for Input quantitation (lanes 1–3). (B) Growth spot assay. Fivefold serial dilutions of WT, *ubp8*Δ, *psh1*Δ, *doa1*Δ, and *ubp8*Δ*doa1*Δ strains were plated on YPD-DMSO and YPD-benomyl (12 and 18 μg/μl, respectively) and grown for 2 d at 28°. (C) The same procedure as in (A) was carried out with, respectively, WT (2); *ubp8*Δ (3); *ubp8*Δ*doa1*Δ (4); *ubp8*Δ*psh1*Δ (5); *doa1*Δ (6); and *psh1*Δ (7). Western blot was sequentially hybridized with anti-myc and anti-6His antibodies; 1/20^th^ of total extracts were run separately and hybridized with anti-myc for Input quantitation. YPD, yeast extract peptone dextrose; DMSO, dimethyl sulfoxide.

Polyubiquitination is a complex and versatile posttranslational modification, in which ubiquitin is linked to a specific lysine to form distinct polymers characterized by multivalent and polybranched ubiquitin chains. It has been proposed that many DUBs, such as yeast Ubp12 and Ubp15 or human USP7, preferentially remove monoubiquitin residues from their target substrates (Schaefer and Morgan 2011), thus opposing chain elongation by the E3 ligases. In yeast, Cse4 is ubiquitylated by Psh1 at its C-terminus *in vitro* (Hewawasam *et al.* 2010; Ranjitkar *et al.* 2010). Moreover, it has been reported that Doa1, a regulator of ubiquitin homeostasis, may also contribute to its ubiquitylation (Au *et al.* 2013). Live microscopy has then revealed a *de novo* replacement of Cse4 at the centromere in S-phase, underlying the dynamic behavior of the unstable Cse4p during the cell cycle (Wisniewski *et al.* 2014). These observations and the ubiquitin-mediated proteolysis of Cse4 advocate a precise role for deubiquitylation in Cse4 regulation. Our findings suggest that Ubp8 interacts with Cse4 by acting on its lysines. Remarkably, lack of Ubp8 causes faster proteolytic degradation of the centromeric histone, which is reverted by deletion of the E3-Ub ligase Psh1 (Figure 3). Since our results point to a novel deubiquitylating activity of Ubp8 on Cse4, we wanted to provide an unbiased, biochemical demonstration of the variations in the Cse4 polyubiquitylation pattern. Yeast strains expressing histidine-tagged ubiquitin (PCUP1:6HIS-Ubiquitin plasmid) and carrying Cse4-myc were created and analyzed (Iglesias *et al.* 2010). 6His-Ub-proteins were purified on nickel columns and analyzed by immunoblotting. Upon induction of the PCUP1 promoter (CuSO4+), tagged ubiquitin was efficiently incorporated and retained on a Ni+column. A regular ladder of 6His-Ub-Cse4-myc conjugate, corresponding to 9.5 kDa multimers, was obtained in the WT strain, confirming an efficient purification step (Figure 4A lane 2; Supporting Information, Figure S1, lanes 3 and 7). Protein extracts from a control WT strain lacking 6His-Ub, and WT and *ubp8*Δ strains expressing the tagged ubiquitin, were eluted and run in parallel with a WT total extract (Figure 4, A and C). Absence of Ubp8 induced a clear change in the Cse4-myc ubiquitylation pattern. The high molecular weight ladder corresponding to longer polyubiquitin chains found in the WT strain (Figure 4, A and C, lane 2) was absent in the *ubp8*Δ strain (Figure 4, A and C, lane 3) which, instead, presented a strong increase in di and tri Ub-Cse4-myc oligomers (Figure 4, A and C, lane 3). This finding suggests that Ubp8 removes a Ub oligomer that may correspond to a K48-linked short Ub-chain, which has a recognized role in proteasome degradation (Kulathu and Komander 2012), in full agreement with the proteolysis pattern obtained (Figure 3). Published results suggest that Doa1 might have a role in Cse4 ubiquitylation (Au *et al.* 2013). We therefore reasoned that it may also affect Ubp8 activity. Microtubule shock under benomyl growth is a useful assay to assess centromeric or mitotic defects. Growth spot assays in the presence of the microtubule interfering agent were therefore performed in *ubp8*Δ, *psh1*Δ, *doa1*Δ, and the respective double mutants *ubp8*Δ*psh1*Δ and *ubp8*Δ*doa1*Δ strains (Figure 4B). Deletion of Ubp8, alone or in combination with Doa1, inhibited growth suggesting that Ubp8 and Doa1 may act on Cse4 through two independent pathways. In contrast, deletion of Psh1 and Ubp8 showed amelioration of the growth phenotype, thus emphasizing the epistatic link between Psh1 and Ubp8 previously shown (Figure 2A). To further investigate the effects of these deletions, we analyzed Cse4 polyubiquitylation patterns in the entire set of mutant strains (Figure 4C). In the *ubp8*Δ*psh1*Δ strain, the high molecular weight poly-Ub ladder was almost completely cleared, leaving a minor lasting signal corresponding to the short Ub oligomer (Figure 4C, lane 5). This persistent short ubiquitin ladder may represent a Psh1-independent polyubiquitylation mechanism, whose effector remains to be discovered and that is not fully degraded in the absence of Ubp8. The polyubiquitylation pattern was also analyzed in the *ubp8*Δ*doa1*Δ double mutant strain (Figure 4C, lane 6). Persistency of the short ubiquitin chain indicated that Doa1 is not directly implicated in deposing the Ub-trimer on Cse4 and excluded any putative link between Ubp8 and Doa1. We cannot rule out, however, that Doa1 might work in parallel with Psh1, ultimately contributing to ubiquitin homeostasis. As expected, deletion of Psh1 completely eliminated the Cse4 poly-Ub ladder (Figure 4C, lane 7), suggesting that, when the E3 ubiquitin ligase is not present and the long poly-ubiquitin mark is not deposed, Ubp8 processes the residual short Ub-chain. The increase in an ubiquitin oligomer obtained in the absence of Ubp8 directly indicates that SAGA DUB Ubp8 is responsible for its processing and removal from Cse4. The absence of a high molecular weight poly-Ub ladder in the *ubp8*Δ-deleted strain further implies that the presence of a Ub oligomer may interfere with and impair the deposition of additional ubiquitin molecules and prevent the formation of longer poly-Ub chains by Psh1 (Figure 4D).

### Ectopic localization of CenH3-Cse4 is enhanced in *ubp8*Δ strain

**Figure 5:**
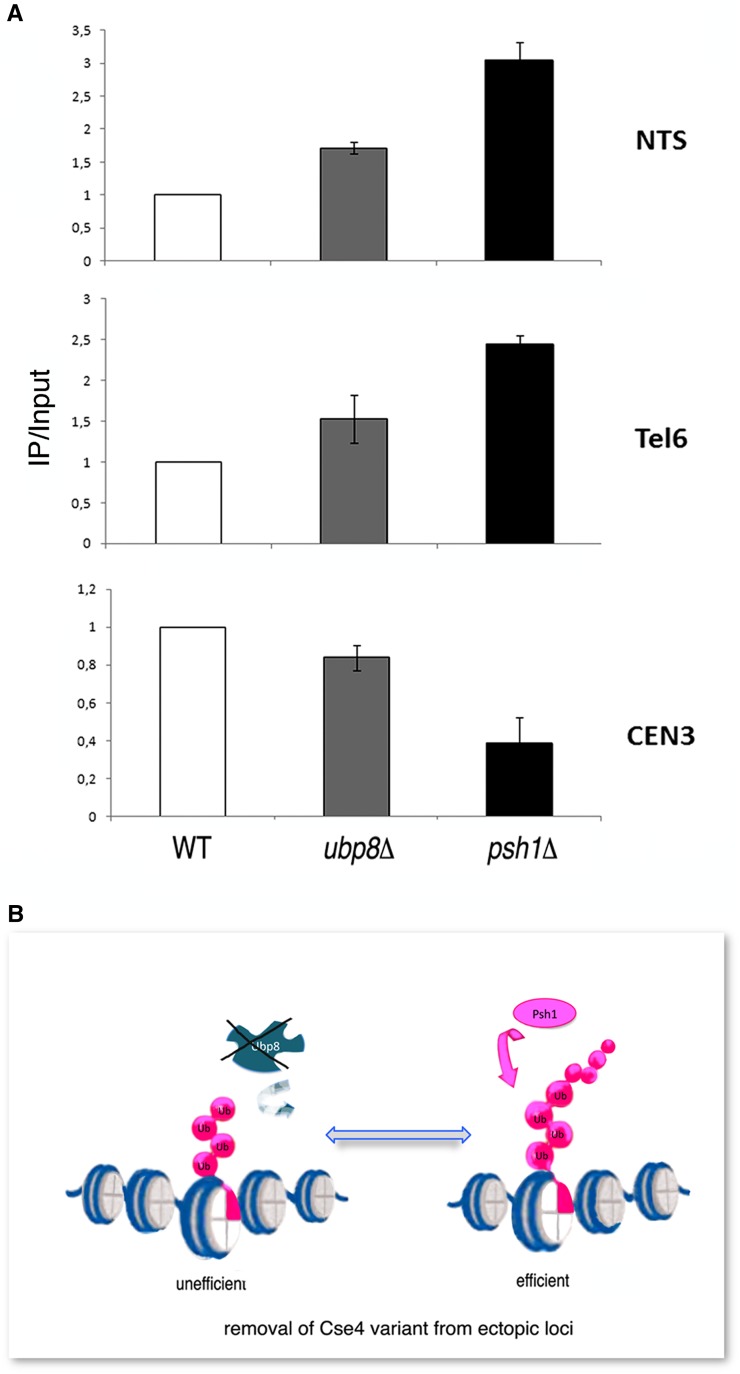
Loss of Ubp8 increases mislocalization of Cse4 at rDNA-NTS and Tel6 regions. (A) ChIP, followed by qPCR, was performed to analyze Cse4-myc localization in wild-type (WT), ubp8Δ, and psh1Δ strains in order to determine Cse4 localization at the centromere (Cen3) and at the ectopic sites rDNA-NTS and telomere Tel6. The values on the y-axis are arbitrary units and represent the fold difference between the amount of target sequence in the immunoprecipitated fraction and the amount of target sequence in the input DNA (IP/Input). Standard error of the mean is shown. (B) Model depicting inefficient removal of Ub-oligomer-Cse4 from ectopic loci in the absence of DUB-Ubp8.

Cse4 is temporarily dislodged from the centromere in early S-phase and its loading is dynamically regulated (Zhou *et al.* 2011). The ubiquitin chain represents the recognition signal for Cse4 removal, which is fundamental in avoiding dangerous misincorporations outside of the centromere (Hewawasam and Gerton 2011). Consequently, in the absence of E3-Ub ligase Psh1, overexpressed Cse4 is increasingly found at ectopic chromosomal loci, underlining the role of ubiquitin in CenH3 restricted localization (Hewawasam *et al.* 2010). Based on our collected results, we decided to assay whether the presence of the short Ub-oligomer accumulating in the absence of Ubp8 might be able to affect CenH3-Cse4 localization on the chromosome. Chromatin immunoprecipitation (ChIP) was performed in WT, *ubp8*Δ, and *psh1*Δ strains carrying an integrated copy of myc-tagged Cse4 expressed at physiological levels (Figure 5A). Chromatin was extracted, purified, and immunoprecipitated with an anti-myc antibody. Quantitative real-time PCR was employed to investigate Cse4 distribution at the centromeric locus CEN3, and at the two noncentromeric sites rDNA-NTS (ribosomal region) (Camahort *et al.* 2009) and Tel6 (telomere). ChIP analyses were carried out in at least three biological replicates. In each experiment, amplification values obtained for each primer pair in the WT strain were used to normalize the values obtained for the *ubp8*Δ and *psh1*Δ strains. WT values were therefore set equal to one. Our results showed an increase in Cse4 localization at the ectopic sites rDNA-NTS and Tel6, both in the *ubp8*Δ and *psh1*Δ strains compared to WT (Figure 5A, NTS and Tel6 panels). The increase of ectopic localization in the absence of Cse4 ubiquitylation in a *psh1*Δ asynchronous cell population had been previously reported (Hewawasam *et al.* 2010), while it had never been described in association with the deletion of the ubiquitin protease Ubp8. We have shown that Ubp8 is required for the removal of a short Ub-oligomer on Cse4 (Figure 4A), which counteracts further polyubiquitylation by Psh1 (Figure 4C). Collectively these findings suggest that the short Ub oligomer, accumulating in the absence of Ubp8, represents a poor signal for the efficient removal of Cse4 from ectopic sites, enlightening a novel step in the control and regulation of CenH3 localization at the centromere in budding yeast (Figure 5B).

## Discussion

Centromere identity is epigenetically defined by targeted and exclusive recruitment of a specialized histone variant, Cse4, in budding yeast. Polyubiquitylation of Cse4 by the E3 RING finger ligase Psh1 has been widely studied, and has been recognized as a central mechanism ensuring the localization of Cse4 at centromeric regions and its removal from ectopic sites (Hewawasam *et al.* 2010; Hewawasam and Gerton 2011). The peculiar lack of any mitotic defect induced by deletion of Psh1 led us to hypothesize the existence of additional processes involved in Cse4 regulation that were yet to be discovered (Folco and Desai, 2010). Therefore, the aim of our study was to elucidate the effects of deubiquitylation, its underlying mechanism, and its putative role in centromeric histone deposition and turnover. In *S. cerevisiae*, the SAGA complex DUB subunit Ubp8 is implicated in the deubiquitylation of histone H2B (Henry *et al.* 2003). The human ortholog Usp22 has been shown to be able to act on other substrates, including SIRT1 (Lin *et al.* 2012). In this investigation, we report the novel finding that Ubp8 is implicated in the deubiquitylation of the centromeric histone variant Cse4. We found that deletion of Ubp8 and Psh1 genetically interacts with a *cse4-1* mutation, that Ubp8 interacts with Cse4 at the physical level, and that its loss induces high mitotic instability, as shown by an ARS/CEN minichromosome assay. We demonstrated that Ubp8 selectively acts on Cse4 lysines by showing that its deletion is lethal if wild-type Cse4 (pGAL-Cse4) is overexpressed, but that it becomes ineffective on an arginine substituted version pGAL-Cse4^K16R^ or when Cse4 ubiquitylation is abrogated by deletion of the Ub-ligase Psh1. The results obtained from the proteolytic profile of Cse4-myc (Figure 3) support these findings and show faster Cse4p proteolysis in the *ubp8*Δ strain, rescued by the subsequent deletion of Psh1. The lethal overexpression of pGAL-Cse4 in the Upb8-deleted strain can be retrospectively attributed to an increased amount of an altered Cse4 version marked by a short Ub-chain (3/4mer) as shown in Figure 4. We can hypothesize that the persistence in the cell of an anomalous version of Ub-Cse4 may also affect its function. Polymerization of polyubiquitin chains is affected by the choice of the lysine linkage that ultimately determines its structure. The variety of ubiquitin chain versions represent not only a signal for the proteasome but also a versatile three-dimensional code, controlling many different cellular functions (Heride *et al.* 2014). There is still a great debate regarding the precise role of Ub-chain species, how their different chain length and linkage type can affect the way DUBs process them, and the meaning and translation of this ubiquitin code into biological processes (Shekhawat *et al.* 2014). The investigation of the mechanisms and the players involved represent a great challenge for the future. Between the vast array of poly-Ub chains that can be assembled on the seven ubiquitin’ lysines, for example, K63 poly-Ub is not a substrate for proteolysis, as reported for NF-κB (Chen and Chen 2013), while a minimal chain length at K48 signals for degradation (Thrower *et al.* 2000; Braun and Madhani 2012). In this study we aimed to demonstrate a novel role for the Ub-protease Ubp8 in the deubiquitylation of Cse4, an epistatic link between E3Ub-ligase Psh1 and Ubp8, and the involvement of this process in centromere function. We have provided an unbiased demonstration at the biochemical level that, in the absence of Ubp8, Cse4 is marked by a short Ub-oligomer. Our analysis reveals a variation in Cse4 ubiquitylation and in the length of ubiquitin chain upon Ubp8 deletion. We also observe that, in the *ubp8*Δ*psh1*Δ strain Cse4, polyubiquitylation is almost completely cleared, strengthening the epistasis between Psh1 and Ubp8. Based on our proteolysis data, we suggest that the short Ub-oligomer accumulating on Cse4 in the *ubp8*Δ strain may likely correspond to a Ub-K48 chain that does not preclude proteolytic degradation (Sadowski and Sarcevic 2010). We propose that DUB Ubp8 is implicated in removing this short Ub-oligomer and we speculate that, in the presence of the short Ub chain, Psh1 activity or subsequent rounds of ubiquitin deposition may be impaired. Indeed, it has been reported that Ub-K48 is able to block further chain elongation on Met-4 (Flick *et al.* 2004). The accuracy of variant nucleosome incorporation at the centromere is of fundamental importance in the control of balanced chromosome segregation in mitosis. Therefore, CenH3 mislocalization is both dangerous and has to be avoided. We asked whether the anomalous short Ub-oligomer accumulating on Cse4 in the absence of Ubp8 might affect its deposition across the chromosome. Our ChIP data clearly show that the short Ub-oligomer is not very effective in signaling Cse4 removal and, accordingly, it increases localization at ectopic positions. Collectively, we demonstrate, for the first time, a novel function of the SAGA DUB subunit Ubp8 as a Ub-protease involved in the removal of a short ubiquitin chain on Cse4 affecting its proteolysis and deposition. A future challenge will be to investigate whether the short Cse4 Ub-oligomer may also be engaged in other epigenetically regulated pathways.

## Supplementary Material

Supporting Information
