# Supplementary material for: SAGA DUB-Ubp8 Deubiquitylates Centromeric Histone Variant Cse4
Source: G3 (Bethesda). 2015 Nov 25;6(2):287–98. doi: 10.1534/g3.115.024877 (PMC4751549; doi:10.1534/g3.115.024877)
Supplement: Supporting Information [file supp_6_2_287__index.html]

SAGA DUB-Ubp8 Deubiquitylates Centromeric Histone Variant Cse4 — Supporting Information 

# SAGA DUB-Ubp8 Deubiquitylates Centromeric Histone Variant Cse4

## Supporting Information for Canzonetta *et al.*, 2016

**Files in this Data Supplement:**

- Figure S1 - (A) Controls for the analysis of 6HISUb Cse4-myc ladder shown in Fig. 4. Protein extracts from WT strains with or without myc tag and 6His-Ub plasmids were purified on a Ni+ column; the eluates were sequentially hybridized with anti-myc and anti 6His antibody; 1/20th of protein samples were loaded and lysate samples were probed with anti-myc as input and anti-Ada2 for internal loading control. (.tif, 6,355 KB)
