# Supplementary figures and images for: SAGA DUB-Ubp8 Deubiquitylates Centromeric Histone Variant Cse4
Source: G3 (Bethesda). 2015 Nov 25;6(2):287–98. doi: 10.1534/g3.115.024877 (PMC4751549; doi:10.1534/g3.115.024877)

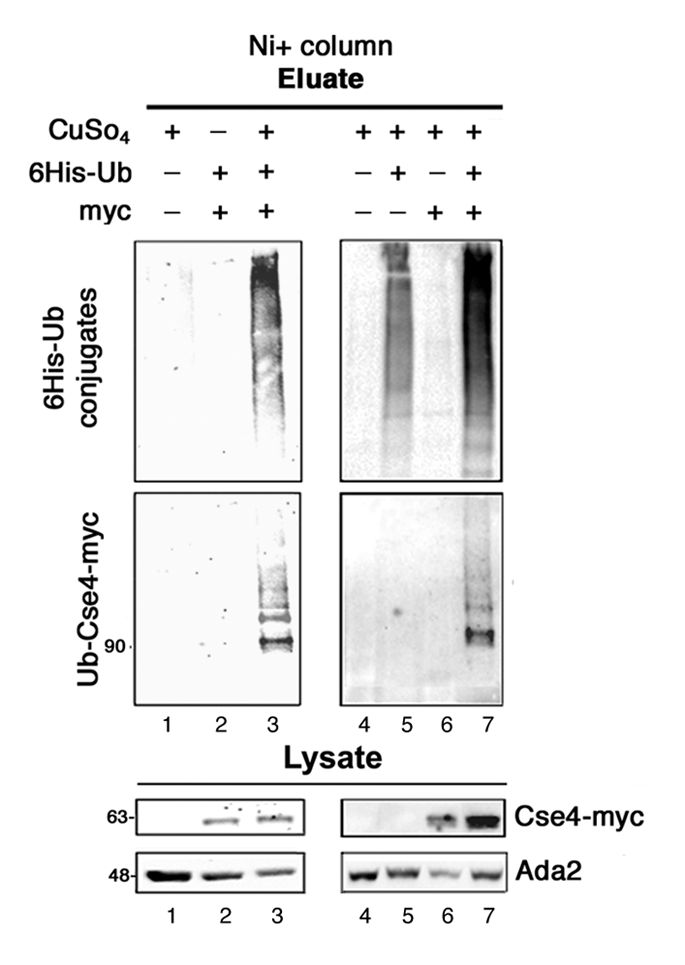

Supplement: Supporting Information [file supp_g3.115.024877_FigureS1.tif]
